# Ferrous Industrial Wastes—Valuable Resources for Water and Wastewater Decontamination

**DOI:** 10.3390/ijerph192113951

**Published:** 2022-10-27

**Authors:** Ecaterina Matei, Andra Mihaela Predescu, Anca Andreea Șăulean, Maria Râpă, Mirela Gabriela Sohaciu, George Coman, Andrei-Constantin Berbecaru, Cristian Predescu, Dumitru Vâju, Grigore Vlad

**Affiliations:** 1 Faculty of Materials Sciences and Engineering, University Politehnica of Bucharest, 313 Splaiul Independentei, 060042 Bucharest, Romania; 2 ICPE Bistrita, 7 Parcului Street, 420035 Bistrita, Romania

**Keywords:** ferrous wastes, mill scale, TiO_2_ production, ferrous sulfate, magnetite, coagulants, adsorbent

## Abstract

Ferrous waste by-products from the metallurgical industry have a high potential for valorization in the context of the circular economy, and can be converted to value-added products used in environmental remediation. This research reviews the latest data available in the literature with a focus on: (i) sources from which these types of iron-based wastes originate; (ii) the types of ferrous compounds that result from different industries; (iii) the different methods (with respect to the circular economy) used to convert them into products applied in water and wastewater decontamination; (iv) the harmful effects ferrous wastes can have on the environment and human health; and (v) the future perspectives for these types of waste.

## 1. Introduction

Wastes can be quantitatively reduced, e.g., through recycling or reusing; often, they can be reintroduced into the circuit from which they came from or are sent to other destinations. When these solutions are not viable, storage and incineration are alternatives.

In the industry, there are materials based on metals that, thanks to the exposed properties, are applied in various fields. To the same extent, waste results from these destinations of the materials, depending on the processing [1]. In 2020, the European Union (27 member states) generated a total of 2.15 billion tons of waste, with the following sectors contributing: (i) construction (37.1%); (ii) mining and quarrying (23.4%); (iii) processing (10.9%); (iv) water and waste management (10.7%); (v) households (9.5%); and (vi) other economic activities (8.4%) [2].

For example, the global steel wire manufacturing market size was valued at USD 102.98 billion in 2021, and it is forecasted to increase at a compound annual growth rate of 4.7% from 2022 to 2030 [3]. However, given the significant impact of the widespread aggression of the Russian Federation against Ukraine, the forecasts regarding the global steel market consumption have decreased appreciably since then. According to the World Steel Association, the predictions for 2022 were downgraded to 0.4%, taking into consideration the current conflict [4]. Unfortunately, these impacts were felt by breaking the raw materials trades. With respect to this, all of the raw material exports from the steel industry were affected: iron ore, pig iron, coking coal, coke, scrap, etc.

Mill scale (MS), which refers to waste generated from steel industry processes, is abundant in iron oxides: Fe_3_O_4_, Fe_2_O_3_, and FeO [5], and accounts for approximately 2% of wire manufacturing (with 37 million tons of MS worldwide) [4,6].

A significant amount of industrial waste is annually generated from the iron and steel manufacturing industry, while the processing of raw materials is returned in the form of sludge, dust, and scale. Some of the iron, approximately 45–50% included in waste, enters into production by sintering and converter manufacturing. The remaining 50–55% accumulate in deposit and sedimentation ponds or are permanently disposed of outside the companies, which leads to a negative impact on the environment. These types of waste have (in composition) harmful materials, such as lead, zinc, and oils. This is why recycling those materials and reusing useful and valuable parts after separation are of significant concern [7,8].

A special focus is dedicated to iron-containing oily sludge from the upper sediments of sludge deposits. The iron content is maintained here in a proportion of 30–63%. They can, therefore, be considered valuable technological raw materials, but the problem is represented by the high oil content (up to 4%) from the conversion in the sintering furnace. In 2020, over 10.8 million tons of oily bottom sediment sludge were stored in the existent maps.

Through iron- and steelmaking manufacturing, different by-products are generated, such as mill scale, slag, dust, and sludge [9]. As a statistic regarding the by-product generation, on average, 200 kg (in the case of scrap-based steelmaking) and 400 kg (in the case of iron ore-based steelmaking) of by-products are produced per ton of steel [10]. Sludge and dust are captured in the abatement equipment, provided with filters. Once separated from the gases, they concentrate large quantities of iron oxides and some carbon, with possibilities of use for internal purposes [11]. The mill scale is mainly generated during the continuous casting and rolling mill operations in oxidizing media.

An iron oxide layer is formed on the surface of the steel. It can be reutilized as raw material in sintering equipment as well as for pellets and briquettes [8].

Forms in which iron is found, e.g., oxides, oxyhydroxides, and sulfides, can determine the adsorption or immobilization of the pollutants from groundwater and waste [12,13,14].

The industrial tests, which succeeded the laboratory ones, have demonstrated the ability of iron to participate in reduction reactions through which the immobilization of some species of interest, such as Cr and/or U, Tc takes place, as well as the dechlorination of various organic pollutants [15,16,17]. Moreover, zero-valent Fe is a strong reducing agent at ambient temperatures, especially for halogenated species [18]. Furthermore, Fe(II) can act as a reducer for halogenated compounds and reduce Cr(VI) species to a less toxic Cr(III) form [18,19].

Because the technical–economic aspects dictate the implementation of technology, in recent years, there have been attempts to find some solutions in which the materials used are low-cost and the source of obtaining is, for example, a waste. In this way, the concept of a circular economy is respected, and the need for reuse, respectively, regeneration, leads to a reduction in the consumption of natural resources. Local or residual materials, e.g., steel wool, iron-coated sand, sawdust, MS, and modified slag, in general materials considered waste or by-products in the smelting and steel industry or Ti production, were identified [20,21,22]. Moreover, the fertilizer industry generates Fe(III)/Cr(III) hydroxide as waste with reliable adsorbent properties, especially for As [23].

Hybrid anion exchangers based on renewable Fe nanoparticles (as alternatives derived from low-cost materials, and whose costs are compensated by the regeneration capacity), can be successfully applied to both water and sludge decontamination [23,24].

Considering these aspects, this paper presents the main achievements regarding the valorization of ferrous wasted materials as potential new-added-value products for society. Comprehensive literature research was conducted between 2010 and 2022 with the main purpose of identifying the main reuse routes of ferrous wastes under the circular economy concept. The main challenge was the lack of data in recent years regarding ferrous waste potential applications; thus, the research was extended to 1992. If agri-food wastes (e.g., biomass are well-known for their efficient applications in environmental remediation, especially for water decontamination and compost use [25,26,27,28], iron as the main component of ferrous waste was still partially reused; today, the main challenge is the transition from the linear concept of disposal to the circular concept of secondary products conversion into raw materials [1,29,30]. The use of wastes with iron content as raw materials integrated into the circular economy concept was intensively studied for the sintering process, pig iron production, ferroalloys or other steel products, or pigment and cement industry [31]. Moreover, due to the advanced investigations, these wastes could be converted into valuable advanced micro and nanomaterials, such as iron oxides, ferrates, or FeSO_4_, with high potential as adsorbents or coagulants for water and wastewater treatment [8,30,32,33,34,35,36].

Coagulation–flocculation has (proven) effective techniques for reducing concentrations of organic and inorganic pollutants in wastewater [37,38]. There are commercially available coagulants on the market all over the world in the forms of iron and aluminum salts (alum, chloride, or sulfates) and polymers [37,39,40]. The prices of these effective reagents often dictate the pace of their use; in the case of aluminum salts, the amount and toxicity of sludge are considerable and involve expensive disposal methods.

**Figure 1:**
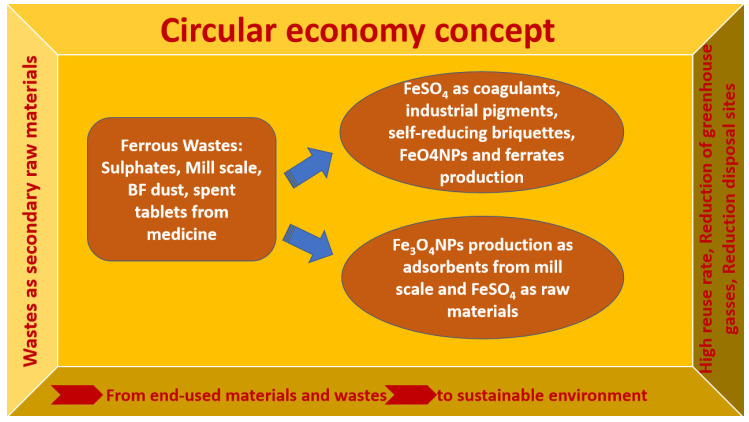
Valorization alternatives for ferrous wastes under the circular economy concept.

Figure 1 represents the main routes of different ferrous wastes valorization in the circular economy concept, with a focus on FeSO_4_ and Fe_3_O_4_ nanoparticles (NPs) as main valuable materials for different applications, with high reuse rates, low greenhouse gasses generation, and a decrease of disposal sites as the most advantageous features.

This research represents a thorough review of the potential applications of the ferrous wastes generated from different sources from the metallurgical industry, with an accent on various conversion and valorization methods for these wastes with regard to the circular economy concepts and efficiency data.

## 2. Iron—An Essential Element for Environmental Equilibrium

The reaction mechanisms involving the Fe species have been intensively studied, most of them being based on the property of exposing a large specific surface area, and, hence, high reactivity, so that applications (such as the purification of mining effluents or those from the textile industry, especially in the retention of As), have demonstrated their efficiency, especially in the formation and use of ferric oxides [34,41,42]. Indeed, the removal of contaminants from waste streams by ferric (hydric) oxide precipitation is an established methodology in a number of industrial processes, for example, the use of high-density sludge systems for arsenic control in effluents from the mining industry, as well as in the treatment of textile dyeing effluents [43].

Iron is an essential element in everyday life. Whether we refer to its biological systems, its ability to bind oxygen, sulfur, or nitrogen atoms, various organic molecules, or its mobility in the natural environment due to the oxidation states in which it can be found (thus, causing sorption and degradation phenomena, especially when precipitation occurs in the form of iron oxyhydroxides), all of these characteristics demonstrate its importance in the balance of life [34].

In the environment, iron has two valence states, which give different characteristics to iron-based compounds: Fe(II) is soluble in water, and Fe(III) is insoluble in water, which is also the predominant form in highly aerated environments at neutral or alkaline pH. There is also a form of iron found under certain geological and environmental conditions, namely zero-valent (or elemental/native) iron (Fe(0)), which rarely forms at the Earth’s surface because its high reactivity causes instantaneous reactions with environmental species and hence changes in valence [44,45,46,47].

In nature, the reactions in which iron participates in the ecological cycle led to the reduction of the polluting potential of many organic, inorganic, and even radioactive contaminants. Inspired by the natural environment, environmental engineers have over time identified various environmental remediation technologies based on processes involving reactions mediated by iron species. Results are well known for soil and groundwater remediation using zero-valent iron nanoparticles for the degradation of organic compounds or the use of iron compounds that generate oxyhydroxides in the natural environment used in the adsorption and/or reduction of As or Cr species [13,48,49,50,51].

One of the adverse effects is represented by the “aging” of Fe species that leads to more controlled structures (such as goethite or hematite) with less active surfaces and adsorption capacities [48].

It exists in decontamination processes, especially through the adsorption of non-oxidized Fe species (sulfides, carbonates, phosphates), which, depending on the pH and redox potential (Eh) values, can act as adsorbents or (co)precipitators for various metal species and radionuclides [48]. An adverse effect, mediated by the presence of Fe species, often encountered, can be observed in the case of mining activities, where there are iron sulfides, and the release into the environment, through chemical and microbial mechanisms in the presence of air and water, of large amounts of solutions strongly concentrated acids (acid mine drainage—AMD), together with metallic species, cause contamination and strong ecological imbalances [52,53].

The presence of Fe species in the soil leads to the release and sequestration of some pollutant species, through microorganisms that reduce Fe(III), which can act by reduction and precipitation on some metals in higher valence states (such as U, Cr, and Tc), both by direct enzymatic reduction and by indirect reduction catalyzed by biogenic Fe(II), at the same time being able to release traces of metals previously bound to Fe(III) phases [54].

For example, in soil, minerals containing iron, in the presence of microbial species, contribute to the reduction of contaminants and the remediation of sites, including in wet areas [52,53,54,55].

The literature indicates two major types of soil and groundwater remediation technologies, mediated by Fe species:

− Sorption and/or stabilization technologies: the use of Fe as an immobilizing agent with the role of adsorbent and/or (co-)precipitant;
− Reductive technologies: the use of Fe as an electron donor for the decomposition or degradation of pollutants to a less toxic or mobile form.

In practice, these types of technologies can be combined.

There are also situations where the applications of Fe-based technologies are effective for less toxic species, such as As(V) compared to As(III), which is why a preliminary oxidation step is initially applied, and the pH is also important, the efficiency decreasing with its increase [21,22].

Iron compounds are present in nature in most deposits of non-ferrous metals, some of the ferrous components being separated during mining. Often, iron accompanies in natural minerals the metallic elements that are the object of extraction, such as FeTiO_3_, CuFeS_2_, or (Ni,Fe)_9_S_8_ [30]. Thus, important quantities of ferrous waste result from hydro- and pyrometallurgical operations.

The risk of damaging natural ecosystems when discharging wastewater with organic and/or inorganic impurities has led to new research in order to identify viable methods. Moreover, it had adapted to the current situation regarding the contamination of aquatic environments with persistent pollutants, combined with the current principles of the circular economy for which the identification of materials with valorization potential contributes to the protection of natural resources.

The well-known methods for iron compounds obtaining follow specific synthesis routes of green chemistry, and new advanced raw materials with multipurpose functions applicable in the treatment and purification of water, as well as for the decontamination of various industrial effluents.

Coagulants with a role in the decontamination of water and wastewater are mostly aluminum sulfates or chlorides, and polyaluminum sulfate and chloride are the most widespread in the water treatment process. The more economical alternative is represented by iron salts, such as ferric chloride, ferric sulfate, or polyferric, as substitutes for aluminum salts [33,56]. Moreover, the mixture of salts from the two mentioned metals can be successfully applied, such as poly-alumino-iron sulfate used to remove coloration and organics dissolved in water [33].

In order to protect natural resources, correlated with Fe behavior in natural environments, and in harmony with waste management and circular economy, the use of Fe sources from industrial ferrous waste, either in the form of ferrous sulfate or transformed into Fe-based nanomaterials, ensures environmental and economic benefits. For example, a source of iron is represented by the waste from the steel industry, which together with Fe ores are integrated into various products in which the addition of Fe is necessary [57,58]. Moreover, iron sulfate heptahydrate (FeSO_4_·7H_2_O) as another ferrous by-product, together with FeSO_4_, could result from the production of titanium dioxide (TiO_2_) can act as a chemical reducer, and raw material for polymeric sulfate and sodium ferrate. In addition, this type of waste has a coagulation efficiency similar to Al salts, and the resulting sludge does not present toxicity, with Fe being an essential nutrient for plant growth [17,59,60].

Iron compounds, due to their essential role in life equilibrium, proved their utility and efficiency in other processes related to life quality, especially in environmental remediation. One of the most efficient and reliable processes consists of using coagulant for water and wastewater decontamination. Generally, the use of ferrous sulfate in industrial coagulation–flocculation processes is preferred to aluminum sulfate, which generates sludge with higher toxicity and implicitly requires more expensive inertization methods [32,37,38]. Coagulation–flocculation remains the most advantageous technique applied in the depollution of liquid effluents of organic and inorganic substances [37,38]. It is important to identify financially and ecologically profitable alternatives, in terms of the type of coagulant applied in decontamination, so that waste and residual materials become attractive [38,61]. Moreover, another alternative to valorizing industrial ferrous wastes involves their eco-friendly conversion into magnetic nano-sized iron oxides as potential adsorbents for water decontamination.

## 3. Industrial Ferrous Waste Sources as New-Valuable Raw Materials for Industrial and Water and Wastewater Treatment Applications

The most representative research studies regarding valorization of industrial ferrous wastes as valuable raw materials in different applications, with preparation and synthesis methods, are presented in Table 1.

According to the presented data from Table 1, two major sources represent potential ferrous valuable raw materials with a focus on FeSO_4_ and Fe_3_O_4_. From TiO_2_ production with FeSO_4_ generation as a by-product from the steel industry, where MS, pickling liquors, scrap iron, and other metallurgical wastes are iron sources, the main characteristics and preparation methods are presented as follows.

### 3.1. Ferrous Sulfate from Titanium Dioxide Production

Ferrous sulfate is an industrial by-product from the cast iron and steel industries and from the manufacturing of TiO_2_. In the case of TiO_2_ production when sulfuric acid is used, more than 7 million tons of ferrous sulfate waste is produced in China every year [65,68]. This type of waste is stored and represents a threat to the environment denoting a blockage in sustainable development and the global circular economy [55]. Being known for its properties in water purification and other applications, methods of its sustainable reuse have been identified in recent years.

Literature indicates that for 1 ton of TiO_2_, approximately 3–4 tons of ferrous sulfate results, together with approximately 8–10 tons of sulfuric acid waste (20%) [68,69,70].

Ferrous sulfate can be used in the manufacture of pigments based on iron oxide, as a chemical reducer or coagulant for water purification, feed additive, areas where natural sources based on iron and sulfur are used, so the reintegration of technical ferrous sulfate in such applications represents an advantage, the market demand being insufficient [17,59,71,72]. Currently, the roasting of pyrite mixed with ferrous sulfate is applied with the production of sulfur dioxide and slag of iron oxides, a disadvantage being the high concentration of SO_3_ in the combustion gases [73]. Another approach for the recovery of iron and sulfur sources is the mixing of waste based on ferrous sulfate and sulfuric acid to generate ferric sulfate oxidation, followed by reduction with pyrite to iron oxide and sulfur dioxide [74,75].

The extraction of TiO_2_ takes place using “chloride” and “sulfate” type processes. In the sulfate process, the raw material is ilmenite (FeTiO_3_), which leads to the generation of amounts of iron sulfate (FeSO_4_·7H_2_O—melanterite) of up to 6 tons of FeSO_4_·7H_2_O per ton of TiO_2_ produced [76]. Relating these figures to the current requirements of the circular economy and sustainable development, ferrous sulfate waste can represent inputs for the synthesis of chemicals and materials [65,77,78,79], such as the use of melanterite in the synthesis of slow-release fertilizers, LiFePO_4_ substituted with cations and for the production of Fe_3_O_4_, through reductive decomposition using pyrite [30].

A recent approach has shown the transformation of industrial iron sulfate into alkali metal ferrates (A_2_FeO_4_, A = Na, K), known as compounds with an oxidation state higher than 3, with applications in various fields, from water treatment as strong oxidizers to bactericidal agents [76]. In an aqueous solution, the ferrous ion (FeOH_2_) is reduced, generating both Fe(OH)_3_ and atomic O. The use of ferrates offers a double advantage, as they can replace chlorine in the water pre-oxidation stage and partly the iron and aluminum salts used as coagulation and flocculation agents. Moreover, the decomposition of alkaline ferrates contributes to the precipitation of heavy metals [80,81,82].

The ferrate synthesis is in accordance with the principles of green chemistry and sustainable development, the products obtained being advanced multifunctional materials.

**Figure 2:**
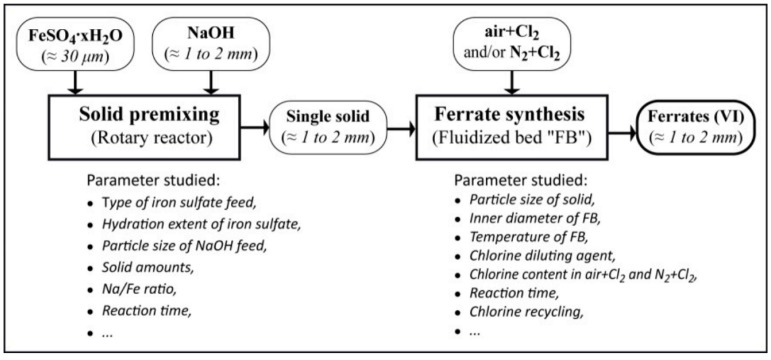
Schematic representation of ferrate production (extracted from [30]).

The synthesis route consists of the premixing of two solids (a base with FeSO_4_·H_2_O), which leads to the coating of iron sulfate in alkaline hydroxides through solid–solid reactions, followed by the actual synthesis of alkaline ferrates in a fluidized bed by oxidizing the product obtained in the first stage with diluted chlorine; the average size of the particles obtained is about 1 mm. The schematic representation of ferrate green synthesis is presented in Figure 2.

The use of FeSO_4_·7H_2_O, as a waste from the titanium oxide manufacturing industry, as a coagulant for the treatment of industrial effluents, is well known. Conclusive results regarding the ability to act as a coagulant were obtained in the case of the purification of secondary effluents from rubber processing processes (SRPE), especially for the elimination of ammoniacal nitrogen (NH_3_-N) and chemical oxygen content (COD) [32]. These effluents generate large amounts of wastewater, about 20,500 L of wastewater/1 ton of rubber, which contain important amounts of inorganic and organic pollutants [39,83]. To increase the efficiency of the studied process, the dose of coagulant (approximately 900 mg/L) and the temperature (62 °C) are two important factors in determining removal efficiencies for NH_3_-N and COD of approximately 98.19% and 93.86%, respectively, after 70 min of coagulation time [32,84]. The advantage of applying this type of waste with coagulant function is, in addition to lowering the concentrations of the analyzed indicators below the limits imposed by the legislation, and the possibility of using the resulting sludge as fertilizer, with a high content of nutrients.

Biological purification, with aerobic and anaerobic digestion, is used for the purification of wastewater from the rubber processing industry, but these methods have limitations [84,85]. That is why the identification of some waste with the role of coagulant represented a viable solution.

Zouboulis et al. (2008) applied FeSO_4_·7H_2_O waste to reduce the biochemical and chemical oxygen content (BOD and COD), solid suspensions (SS), and ammoniacal nitrogen (NH_3_-N), at variable parameters: pH, coagulant dose, time, temperature [59].

Ferrous sulfate from the manufacture of TiO_2_ using the sulfate method leads to the generation of more than 7 million tons of ferrous sulfate waste every year in China, which leads to the storage of a large amount of waste and the increase of environmental protection costs [68,86]. Su et al. (2005) indicated the use of ferrous sulfate to reduce Cr(VI) to Cr(III) during the processing of chromite ore, as a method of valorizing this waste [71].

**Figure 3:**
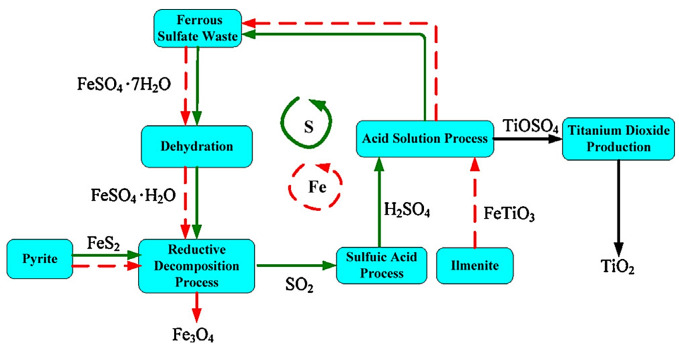
Recycling sulfur and iron from ferrous wastes [65] (with permission from Elsevier, copyright year 2022).

The preparation of polymeric Fe sulfate for water treatment is also a solution, other than the production of industrial pigments or feed additives [59,60,61,62,63,64,65]. However, obtaining a product with added value is restricted by the low demand on the market for iron and sulfur resources, which is why the operation of roasting pyrite with ferrous sulfate is often applied to obtain Fe oxide slag and sulfur dioxide, but the pig iron presents the disadvantage of combustion and SO_3_ in combustion gases that cause corrosion of installations, therefore the enrichment process by roasting pyrite mixed with ferrous sulfate waste is still being studied, there are promising results when introducing some additional oxidation stages [53]. The recycling sulfur and iron process from ferrous sulfate processing was represented by Huang et al., as observed in Figure 3 [65].

The thermogravimetric results of the ferric sulfate decomposition process by reduction with pyrite showed that the final sulfate decomposition temperature was 450 °C, and when the ferric sulfate decomposition took place directly, it was lower, resulting in a gaseous product: SO_2_.

The mechanism underlying the sulfate decomposition by pyrite was solid–solid, with intermediate formation of Fe_2_O_3_. Kinetic studies showed that the Fe_3_O_4_ generation process was continuous [69].

**Figure 4:**
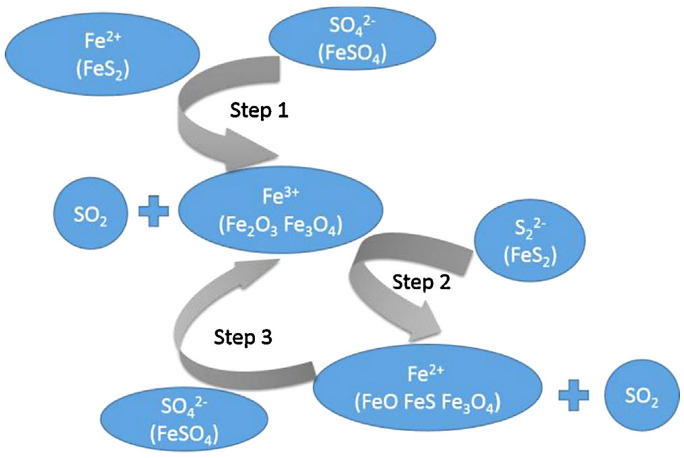
Reaction mechanism of autocatalytic ferrous sulfate decomposition [65] (with permission from Elsevier, copyright year 2022).

Ferrous sulfate decomposition intermediated by pyrite in nitrogen atmosphere indicated as main products Fe_3_O_4_ and SO_2_ [65]. The kinetic data suggested that the decomposition process was based on autocatalytic reactions, with Fe_2_O_3_, FeO, and Fe_3_O_4_ being catalyst components. Figure 4 summarizes the three main steps of the process: (1) heterogeneous reaction between FeS_2_ and FeSO_4_ onto the solid material surface, with Fe_2_O_3_ and Fe_3_O_4_ solid products formation; (2) Fe^3+^ reduction by S_2_^2−^ to Fe^2+^ as FeO, Fe_3_O_4_; (3) SO_4_^2−^ reduction to Fe_2_O_3_ and SO_2_ by FeO and Fe_3_O_4_ [65].

One method of using FeSO_4_·7H_2_O from TiO_2_ production is to obtain magnetite useful in steelmaking. Synthesis methods can be co-precipitation, obtaining microemulsions, sol-gel syntheses, and hydrothermal or solvothermal reactions [87,88,89,90,91]. The cheapest and easiest method of obtaining it is the co-precipitation of Fe^2+^/Fe^3+^ ions (molar ratio 1:2) with sodium hydroxide or ammonia solution [92,93,94], followed by magnetic separation of the formed product [95].

Calcium hydroxide can also be used as an alkaline medium and added to the ferrous sulfate solution as a waste from the TiO_2_ manufacturing industry [95]. Fe^2+^ ions from the reaction solution were precipitated as Fe(OH)_2_ and transformed into Fe_3_O_4_ by oxidation and heating with air. A mixture of magnetite and gypsum is obtained; after wet grinding, it can be magnetically separated in order to obtain pure magnetite.

### 3.2. Mill Scale from Rolling Process

Considering that steel production is an essential activity in society, many of the results regarding the reuse of some waste from this industry were obtained locally, for example in the Brazilian producing companies [63].

In the steel processing operation, during continuous casting and hot rolling, a layer of iron oxides, known as mill scale, forms on its surface. Its removal takes place by spraying with water, and this mixture of iron oxides is reintroduced into the circuit or utilized by different methods. Within the semi-integrated steel mills, there is also a large amount of waste that requires reuse/recycling/reintegration into the circuit. The most important, from a quantitative and compositional point of view, are: electric arc furnace dust, slag, sludge, and MS. The correct management of the assets in the direction of valorization offers benefits to the environment and society [63,96].

MS results from the hot rolling process of steel, being a mixture of iron oxides and metallic iron, with variable oil and fat content. It is estimated that the production involves about 10–20 kg/t of steel production [63]. This type of waste can be processed and integrated into concrete or bricks, resulting in ecological materials, e.g., similar to the pavement applied in parking lots. It can also be a raw material for self-reducing briquettes in the electric arc furnace and the manufacturing of coagulants, such as ferrous sulfate or iron sulfate.

The results obtained from the conversion of MS into ferrous sulfate, by leaching with sulfuric acid, without applying complex techniques, are promising at the laboratory level, but have not yet been transferred to the industrial level until now.

Melanterite (FeSO_4_⋅7H_2_O—ferrous sulfate heptahydrate) is a commercial form of ferrous sulfate and it can result as waste in the production of TiO_2_ and the pickling of steel with sulfuric acid; it can be used directly in water purification processes, with the coagulant role. The product has another use—that of a component in medicines used to combat anemia (both in humans and animals).

The literature indicates bio/hydrometallurgical routes for the production of ferrous sulfate heptahydrate from coal tailings and MS as raw materials [63,97].

MS represents a source of iron, composed mainly of magnetite, with a porous appearance and hard surface, in which the iron content can reach about 93% [67]. Commercially, this mixture of iron oxide, predominantly with magnetite, combined with nickel and zinc oxide can be used to manufacture soft magnets, components of audio–visual devices. Currently, MS production of elemental Fe is an energy-intensive process. In addition, in the case of micro-Fe formation, it does not show a high reactivity to produce hydrogen, and the process also leads to the formation of CO_2_. A transformation into nano-scale Fe is indicated by Kesavan et al., implying the obtaining of some reactive structures. MS as a precursor of nano-zero valent Fe (nZVI) production can be converted into nanoparticles using different routes: (i) reductive process with NaBH_4_ for nanoparticles with 30–40 nm diameter; (ii) mild solvothermal method using hydrazine or surfactant-cosurfactant in water–oil microemulsion for 5 nm diameter nZVI NPs formation [67]. Moreover, the iron oxide used after the metal–steam reaction can be utilized by transforming it into nZVI, offering the advantage of hydrogen generation in several cycles [67].

MS iron waste is well-known as a precursor for industrial pigments as iron oxide components. By preparing a ferrous and ferric precursor from acidification of MS at different amounts, magnetite, maghemite, goethite, and hematite are obtained at different temperatures with specific color shades, from red to orange, purple and grey combination with brown, composed of small particles, lower than <0.1 mm, exposing high reactivity due to the high surface area [66].

Moreover, the colored ceramic pastes of the sandstone type can be obtained by incorporating variable amounts of MS, the leachability tests indicating the stability of this waste in the ceramic matrix [1].

Portland cement factories and those that produce various construction materials reuse MS. The advantages would be that by introducing clinker into the raw mass, it contributes to the burning capacity and has resistance to compression [57].

The MS can be reused as a component in clinkers to improve its durability, together with pyrite, limestone, and gypsum [57].

MS, as a raw material for obtaining ferrous sulfate, can be subjected to the leaching process with sulfuric acid, at different concentrations, which can lead to different products based on Fe sulfates, such as melanterite (FeSO_4_⋅7H_2_O), szomolnokite (FeSO_4_⋅H_2_O), and rosenite (FeSO_4_⋅4H_2_O) [63].

### 3.3. Pickling Liquors from Metal Finishing Industry

The metal finishing industry generates significant quantities of pickling liquors, whose composition represents a threat to the environment, due to the toxic components. In this industry, the pickling operation applied to the sheet, strip, or wire products, or parts subjected to galvanizing or painting involves the use of acids or acid mixtures.

The steels are pickled using solutions of approximately 15–20% HCl or H_2_SO_4_, at approximately 100 °C, the process of pickling the oxides on the surface of the steel being repeated in the acid solutions in which chlorides and, respectively, sulfates of Fe(II) are formed, together with other accompanying metals such as Zn, Cr, Ni, etc., depending on the type of steel treated. The strongly corrosive environment determines that these solutions cannot be used or stored, without neutralization that takes place with lime, and the sludge generated is dumped or evaporated and cooled; these operations lead to the crystallization of FeSO_4_⋅7H_2_O [62].

In order to protect the environment, it is recommended to recover and reuse the acid and dissolved iron (II) in order to transform them from waste into materials with added value, usually using tri-butyl phosphate for acid extraction [98,99] or other commercial products based on phosphines or amines [62,100]. In the case of Fe(III) extraction, extraction with solvents, such as methyl isobutyl ketone (MIBK) [101], tri-n-butyl phosphate (TBP) [98,99], di(2-ethylhexyl) phosphoric acid (D_2_EHPA) [102,103,104], and amines [105,106,107,108].

Ferrous waste can also be used in the process of generating hydrogen needed by cells for the fuel that ensures energy transport, with applications in the automotive industry. Obtaining high-purity hydrogen, with a high cost, including for the stages of transport, storage, and transport to users determines the identification of alternative sources, and the iron from these wastes through steam reforming (water vapor) can generate hydrogen [67].

In practice, the iron is integrated into cartridges that may or may not be preheated, the steam added to the cartridges produces pure humidified hydrogen, and the iron oxide from the used cartridge is again transformed into elemental iron.

In the steel industry, pickling operations result in highly acidic concentrated liquors with high concentrations of Fe. These liquid wastes are considered dangerous for the environment, they are neutralized, resulting in high amounts of metals precipitated in the form of hydroxides [64].

In recent years, various methods of ion exchange [109,110], hydrothermal with microwaves [111,112], membrane distillation [113,114], precipitation, and selective extraction [62,115,116], have been applied to reuse metals with valuable potential. The methods were effective in terms of reducing the polluting impact on the environment, also resulting in the concentration of Fe salts and oxides, these representing cheap reagents and offering the possibility of replacing some commercial products intended for various industrial applications with these wastes.

Magnetite (Fe_3_O_4_), for example, is recognized for its use in magnetic storage media, solar energy, electronics, as a catalyst, medical imaging, a contrast agent, but also as a biosensor [117,118,119,120,121,122,123,124,125,126].

The most common method of obtaining Fe_3_O_4_ is the coprecipitation method, in which the particles are obtained on a micrometric scale, and their reactivities are reduced [64]. The continuous development of technologies has led to the need to approach various devices at the nanoscale, especially electronic ones, but also those in biomedicine and biophysics. Thus, sonochemical and ultrasonic methods have been developed, mainly for obtaining Fe_2_O_3_, CdS, Mn_3_O_4_, or ZnS-type nanoparticles with potential for catalysis, luminescence, and various optical devices [127,128,129,130,131,132,133,134,135].

If in the case of coprecipitation a surfactant is necessary [64] in order to have rigorous control of their sizes and distribution, in the case of the sonochemical method [121,136] and Fe precursor is necessary, with a high cost, to obtain the crystalline or amorphous particles of Fe_3_O_4_. As a rule, the precursors used in the sonochemical method are organometallic, these being replaced by solutions used from pickling, concentrated in Fe [137,138,139] in the case of obtaining Ni and Zn complex ferrite, of nanometric dimensions, through the microbial oxidation of Fe(II) to Fe(III), in dilute solutions.

Moreover, a co-precipitation method assisted by the ultrasonic method led to the obtaining of magnetic ferrite nanoparticles from the precursor solution used from steel pickling [64]. Moreover, the ultrasonic-assisted chemical co-precipitation method of spent pickling liquors leads to cubic Fe_3_O_4_ nanoparticles production, with 13–23 nm diameter and super-paramagnetic behavior, homogenous size, and shape distribution, features obtained by an environmentally friendly and simple approach [64].

### 3.4. Other Metallurgical Wastes

Another type of ferrous waste in the metallurgical industry is furnace dust, from which self-reducing briquettes can be obtained at high temperatures and in a reduced atmosphere [6]. In this way, this waste could be reintroduced into the circuit, in low-height furnaces, together with MS, thus contributing to the sustainability of the steel industry.

Regarding wastewater contamination, Cr (VI) is one of the monitored pollutants due to its high toxicity, both to humans and animals, but also to its mobility in the soil, so it can also appear in groundwater [140,141]. The less toxic form, Cr(III), precipitates more easily in the form of Cr(OH)_3_, but also in combinations with Fe(III) in the form of oxy(hydroxides), at alkaline or slightly acidic pH [142,143,144,145]. Due to the low solubility at the natural pH of the waters, it is often found below the maximum limits allowed by the legislation. The methods regarding the decontamination of Cr (VI or III) have been intensively studied, the ones involving the reduction of Cr (VI) to Cr(III), followed by precipitation, as well as adsorption, membrane separation, and bioremediation are known to be highly efficient [146,147]. For the reduction method in which the highly toxic hexavalent state is reduced to Cr(III), ferrous sulfate, sulfur dioxide, and sodium sulfite are used. With the development of nanotechnologies, the use of nano-zero valent Fe is applied in situ in redox reactions of active metals present in the form of groundwater contaminants [144,148,149].

The reuse of scrap iron and its recycling for the production of Fe powder, reinforcing agent, or Cr (VI) reducing agent during galvanic pickling has been studied and applied at the industrial level as well [150,151,152,153]. The reduction of Cr(VI) from water have been less studied, but the results are promising in the case of using steel wool [154]; Gheju et al. studied the reduction of Cr(VI) by using local scrap iron, as a cheap, available reducing agent, which at low pH values, a temperature of about 40 °C, and a low concentration of Cr(VI) (about 19 µM) shows high reduction speeds up to when the passivation of the old Fe surface takes place by forming a mixture of (oxy)hydroxides of the Fe(III)-Cr(III) type [149].

### 3.5. Acid Mine Drainage

The process of acid mine drainage (AMD) represents a threat to natural waters; the treatment involves the addition of alkaline reagents to precipitate metals in the form of hydroxides with an increase in pH values [33]. The operating costs regarding the removal and treatment of the produced sludge represent another disadvantage, although the remediation process has high efficiency [155,156,157]. Considering the content of about 2–5% solids, together with high concentrations of iron and aluminum and low concentrations of manganese, and zinc, in order to reduce the negative impact on the environment, the recovery of valuable elements from these sludges in the form of hydroxides is a priority, especially for the production of coagulants [158], ferric oxide nanoparticles, and inorganic pigments [56,159,160,161,162].

An example can be the production of ferric sulfate from the acidification of ferric hydroxide from AMD precipitation using amine to eliminate interferences and keep the pH between 3.5 and 3.6 [158].

Iron and aluminum could be recovered from AMD waste in a proportion of 97% and 98%, respectively, through a process of oxidation and selective precipitation, obtaining a coagulant rich in aluminum and iron, in the form of a concentrate after the solid separation operation–liquid by centrifugation and/or filtration, and dissolution in sulfuric acid, at pH 5.

### 3.6. Other Ferrous Wastes

Two types of waste can affect the quality of the environment through storage and have the potential for combined reuse, namely expired drugs containing ferrous sulfate granules and used Li-anode batteries in the form of foils. Simultaneous recycling through hydrothermal treatment leads to obtaining LiFePO_4_/C powders [35]. Medicines containing ferrous sulfate are intended for patients with anemia, and in addition to the active ingredient, they also contain ascorbic acid and glucose. However, often the demand is greater than the consumption itself, resulting in waste from hospitals, pharmacies, and households, which are collected and incinerated, in the case of households being mixed with garbage [65,163]. Thus, in accordance with the principles of the circular economy, the valuable potential of these expired drugs, which have become waste, can be exploited, replacing the natural resources of Fe.

Moreover, Li-based batteries used for energy storage and conversion, after end-of-use, lead to the formation of Li foil-type metal waste [76]. The lack of adequate recycling of these leads to the risk of fire due to the relatively low melting temperature of Li (180 °C) through accidental overheating, in addition to environmental aspects [164]. Li resources are rare, so the identification of solutions regarding the reuse/recycling of this element is necessary. In this way, the simultaneous recycling with ferrous sulfate granules from expired drugs, applying the hydrothermal method, leads to obtaining nanosized powders (60–400 nm) of the LiFePO_4_/C type for the cathode in lithium-ion batteries, thus obtaining a material with added value at a low cost and a negative impact on the environment, presenting stability up to 500 cycles for higher rates of 5 °C and 10 °C and the reversible discharge capacities being 123 and 94 mAhg^−1^, respectively [59].

## 4. Performances within Iron-Based Coagulants and Adsorbents from Ferrous Wastes in Water and Wastewater Treatment

The main application of ferrous sulfate for environmental remediation is related ot water and wastewater treatment when FeSO_4_ (as a coagulant) could be used as solid waste or after pretreatment methods. Moreover, its use as a precursor for magnetic nanoparticles with adsorption and degradation properties for heavy metals and organics offered new perspectives on the valorization potential of this ferrous waste.

**Figure 5:**
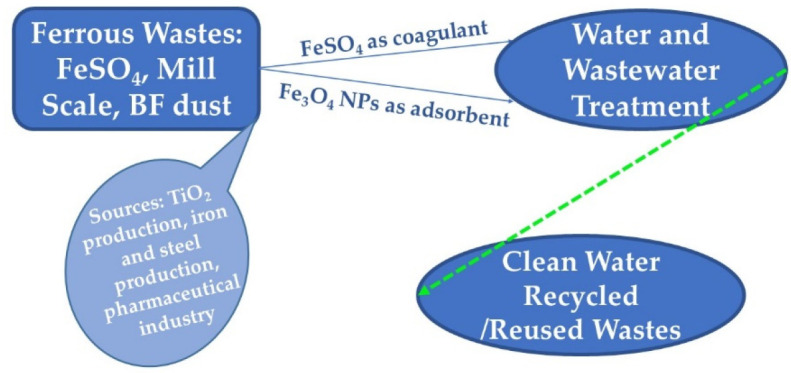
Ferrous wastes and their product applications for water and wastewater treatment.

As a synthesis of iron compounds from industrial sources, with advanced properties, Figure 5 depicts the main ferrous waste sources and their water and wastewater treatment applications.

FeSO_4_ from TiO_2_ production was tested as advanced material integrated into the leaching tests applied to air pollution control residues (APC). The experiment is known as the Ferrox treatment process, where 15 g Fe^2+^/kg fly ash (FA) and 60 g Fe^2+^/kg semidry APC-residue (SD) were brought into contact for 24 h of stirring and aeration, at a liquid–solid ratio (L/S) of 3 L/kg. The experiments indicated a good capacity of binding for pollutants, such as heavy metals and salts. In this way, the leachate contained small concentrations of pollutants due to the iron oxide formation onto the APC surface that retained the metals [165].

Mohammad Ilias et al. indicated that FeSO_4_⋅7H_2_O waste from the titanium industry represented an effective coagulant for secondary rubber processing effluent (SRPE), with high efficiencies for ammoniacal nitrogen (NH_3_-N) and chemical oxygen demand (COD) removal [32].

FeSO_4_⋅7H_2_O waste exposed coagulant properties that sustained its sustainable utilization and generated zero waste and environmental protection.

Moreover, from acid coal mining drainage liquors by chemical precipitation could be obtained an efficient coagulant, such as poly-alumino-iron sulfate, which, in comparison with coagulant from iron scrap or conventional aluminum sulfate, has almost the same efficiency at a low cost and sustainable approach, almost 98% of generated waste as sludge being reduced by this utilization [33].

The main performances reported while using different valorized ferrous wastes are reviewed in Table 2.

Ferrous wastes, such as steel wool, cast iron filings, iron-coated sand, and blast furnace slags can be integrated into filter designs, especially for As removal [20,21,22,34]. Moreover, regenerable iron nanoparticle-based hybrid anion exchangers could be efficient water treatment materials synthesized from ferrous wastes [24,34].

Referring to ferrous wastes used as raw materials in water treatment, many studies were developed on As removal, the pentavalent (As(V) or arsenate) state being more studied, even if the more dangerous state is trivalent (As(III) or arsenite) state, often in the process being implicated an oxidation step as pre-treatment [21].

For example, cast-iron filings (wastes from mechanical workshops, lathes) and steel wool (commercially available, used for cleaning wood surfaces prior to polishing) could be involved in As adsorption process as low-cost materials derived from wastes, when the efficiency is almost 90–95% for As removal [21].

Another type of waste is Fe(III)/Cr(III) hydroxide. This can be obtained by the precipitation of Cr (VI) compounds, which resulted as corrosion inhibitors for cooling water systems and electrolytically resulted Fe(II), which reduces Cr(VI) to Cr(III) under acidic pH, followed by precipitation with lime as Fe(III)/Cr(III) hydroxides [22]. The adsorption capacity was studied for As(V) and the process was explained by pHpzc of hydroxide surface (8.3) responsible for H_2_AsO_4_^−^ adsorption species (in pH range 3–7) due to the Coulombic interactions [22,23].

MS waste combined with a lithium-ion battery (LIB) anode led to graphite-supported zero-valent iron–copper bimetallic catalysts (ZVI-Cu/C) formation by carbothermic reduction. These types of catalysts obtained from two types of waste have good degradation efficiency for 4-CP in water [167].

The supposed process involves reduction and heterogeneous Fenton reactions. Moreover, regarding the low leachability of Fe and Cu, there is a recycling option for the catalyst; the results indicate that the possibility of applications for other pollutants is degraded by oxidation and that a reduction could take place.

## 5. Ferrous Wastes Perspectives

Solid waste management is an international concern, especially with the increase in consumption and the limitation of natural resources.

Good management means future solutions for economic well-being and a high-standard life, in which care for the environment, nature, and the use of ecological products are priorities.

Uncontrolled, massive storage, or accidental spills directly affect water, air, soil, and the quality of life.

Iron oxides can be the bases for industrial pigments with applications in paints, coatings, enamels, and plastic materials [1,169]. It has been observed that for each type of paint there is an optimal amount of MS in order to preserve the anticorrosive capacity (1 to 15% by weight) [169]. Moreover, the mechanical and thermal pretreatments of MS and specific added quantities (1 and 10% by weight) influence the intensity of the brown shade of the glaze.

In the ceramic industry, the shades provide aesthetic effects for the surfaces of the pieces, and the use of the same glazes simplify the production process for colored pastes, thus reducing the time needed to change them.

In 2019, the steel requirement reached approximately 1.9 million tons, but in the first quarter of 2020, there was a considerable decrease of approximately 20%, the sector being impacted by the global situation regarding the pandemic [170] target for 2050 is a production of about 2.8 million tons in 2050 [6,171].

Steel is 100% recyclable, fitting into the circular economy concept, it is environmentally friendly due to the durability of this material, with the European steel industry being a market leader.

However, it is estimated that approximately 600 kg of waste (dust, sludge, MS) is generated per 1 ton of steel produced [172]. One plus is the possibility of recycling or reintegrating into other industrial sectors, but there is also a stored amount. The advantage of recoiling can be seen in the cost price of steel, and about 48% of steel production in the EU is based on scrap metal; in coming years, considering the reduction of carbon emissions, the focus will be on recycling in the steel industry [173].

The EU also exports waste from this industry to third-world countries, with scrap metal representing about 48% of all recyclable materials exported (about 19.5 million tons in 2021) [174].

Waste generated in the production of iron and steel is MS, where the iron content is about 2% of the total steel produced [175]. MS is considered from the point of view of its physicochemical composition to be on the list of green products, being a non-hazardous waste, according to EU legislation regulations [31]. Its formation takes place during the continuous casting of steel in the rolling stages, when, due to thermal gradients and the oxidizing environment, a layer of iron oxide grows on the surface of the steel, consisting of gray–black Fe_3_O_4_ magnetite in the form of fine metal grains, with FeO particles, and on the outside a thin layer of hematite Fe_2_O_3_. The dimensions of these solid particles are about 10 mm.

Moreover, this blast furnace waste dust is an important source of iron and carbon oxides, resulting in about 10 kg/ton of material [176].

In the case of integrated combines, MS can be recirculated during sintering, while BF dust, due to its very fine particles, has a limited recycling capacity [175,177]. The recycling of iron from oxides and carbon can be achieved by forming self-reducing agglomerates in the form of composites from powdery waste [178,179]; the reducing agent is carbon that reduces iron oxides to iron metals.

Thus, MS and BF dust can be ideal candidates for the formation of composites as briquettes; however, the data about them are quite scarce, especially regarding their mechanical resistance.

Bagatini et al. reported such results using MS and BF dust for the shaft of the low-height furnace, with the realization of a mass balance and the recycling of these wastes in the context of the circular economy [6].

Briquettes with the highest amounts of MS combined with BF dust are the best combination for low-height furnaces that do not operate under severe conditions and have low material strength requirements.

The cleaning steps applied to the finished products in the stainless-steel finishing process led to the removal of dust, limescale, and metal oxides [66]. From these cleaning stages, the scale of the mill also results, after the hot rolling of the steel, in the form of a mixture of iron oxides and metallic iron, with a variable content of oil and fat [66]. It is estimated that the result is about 35–40 kg/t of the hot rolled product. An alternative to recycling is to introduce it into the sintering process, but after removing the oil, an alternative that involves costly pretreatment processes to remove it and has a negative impact on the environment. The introduction of MS in the sintering process can take place at oil concentrations below 1% and sizes of about 0.5–5 mm for the oxide mixture. Above 3% oil, volatile organic compounds are released, including dioxins [84].

When the MS particles are below 0.1 mm, they absorb oil in a proportion of about 5–20% and its reintroduction in the sintering process cannot take place. Oil absorption is beneficial when the reuse of Fe oxides in the form of industrial pigments is desired, the demand on the market being high, especially in construction activity [180,181]. They do not present toxicity, have low costs, are durable, and are chemically stable. In order to avoid the storage of such waste, the literature indicates an effective solution is to convert it into materials with an adsorbing role in water decontamination.

The oxides used as pigments are based on magnetite for black, hematite for red, maghemite for brown, and goethite for yellow [66,84,180]. Moreover, by decomposition at different temperatures, the waste formed during the pickling of steel can lead to the formation of red hematite. During the formation of MS, corrosion products also appear in the form of a mixture of Fe oxide products; the color is weak, the pigment value is reduced, the surface of the metal is mainly covered with FeO, and the exterior with Fe_2_O_3_, as well as the existing Fe_3_O_4_ and FeOOH [182]. Data from the literature indicate the obtaining of magnetite from MS by dry oxidation, and then by calcination, hematite could also be obtained, in both cases, the particles were coarse and required grinding below 10 mm to meet the pigment requirements and ensure color resistance, covering power and oil absorption capacity. Legodi et al. demonstrated that, from MS precursors, soluble in water, magnetite (black), hematite (red), goethite (yellow), and maghemite (brown) can be obtained as homogeneous pigments, porous and with good properties, with purity and corresponding dimensions (below 0.1 mm) [66]. The color shades were obtained depending on the thermal treatment applied.

Waste incineration is today applied as a final solution to reduce the negative impact on the environment. It is important to note that with the control of gas emissions, at the chimney, there are different types of equipment, such as electrostatic precipitators, and wet, dry, and semi-dry scrubbers to reduce the generation of dusty waste that is considered dangerous. In particular, in countries such as Denmark and the Netherlands, these dusty wastes are closely monitored, especially due to the high concentrations of salts (Cl and SO_4_ in combination with Ca, K, Na) and heavy metals (Pb, Cd, Zn, Cr, Hg) [165]. The storage of such waste, without pretreatment, leads to dangerously concentrated leachates. In this context, Lundtrop et al. developed the Ferrox process, in order to remove soluble salts in order to avoid complexation with heavy metals and stabilize them in solid form, while maintaining alkalinity to ensure increased buffer capacity in order to avoid acidification of the environment. Thus, the dusty waste is introduced into an alkaline solution of ferrous sulfate (3 L/kg), subsequently forming Fe oxides. Fe(II) changes to hydroxide, in alkaline suspension, later becoming a solid ferric hydroxide, through oxidation, with the ability to bind and stabilize heavy metals [165]. The advantage is given by the stability of these oxides both in terrestrial and aquatic environments.

## 6. Conclusions

This literature research emphasizes the role of ferrous wastes as potential valuable raw materials in water and wastewater treatment and other industrial applications. Even if major iron wastes are valorized today, the use of iron compounds as coagulants and advanced nanomaterials in water and wastewater treatment involves high production costs and the option of ferrous waste reuse could lower these costs.

There are also positive and critical aspects regarding their valorization, for example, if the quantities are sufficient, not all converting processes are completely green or accessible for industrial production.

Usually, the iron and steelmaking industries, as well as TiO_2_ production, are the main sources of ferrous waste production, especially as MS and FeSO_4_. The paper presents some representative examples of waste processing as valuable raw materials and the performances within their use as coagulants or nanomaterials. Moreover, the potential use of pigments is underlined.

The data and results summarized in this paper offer new perspectives in waste management linked with the circular economy, focused on the need for new technological solutions for the quality of life and the environment, where higher by-product qualities increase their valorization options integrated into environmental and economic sustainable concepts. TiO_2_ production (along with the steel industry, where the “zero-waste” goal is proved in a sustainable way) represents an important ferrous waste that is useful for wastewater decontamination. Moreover, natural resources are protected, environmental impacts are reduced, and the production processes have less energy consumption.

Within these considerations, the most important findings of this literature research are based on: (i) a comprehensive presentation of technical solutions regarding the integration of different ferrous wastes into the water and wastewater treatment routes as valuable materials, such as FeSO_4_ and Fe_3_O_4_ nanoparticles (NPs); (ii) the highlighting of industrial sources that generate ferrous wastes, their processing methods, and quantitative data; (iii) the efficiencies and laboratory/pilot tests related to water and wastewater decontamination with an accent on target pollutants.

This study reveals the industrial ferrous waste potential from a new perspective as reliable coagulants and adsorbents with the same characteristics as commercial ones.

In this way, the valorization of ferrous wastes as alternatives for raw materials represents an actual option for today’s consumption and environmental threats.

## Figures and Tables

**Table 1:** Ferrous waste sources and the final products for industrial and water treatment applications.

Waste Source	Waste Valorization Method	Type of Product	Reference
Acidic ferrous solutions from steel pickling	Extraction with binary solvent (methyl isobutyl ketone—MIBK) and (di(2-ethylhexyl) phosphoric acid—D_2_EHPA).	Fe(III) extracted from concentrated iron solution in a sulfate medium.	[62]
Coal AMD	Oxidation/selective chemical precipitation, solid–liquid separation, 24 h aeration, pH 2.5–3.0 (conversion of Fe^2+^ to Fe^3+^), 4 M NaOH, precipitation of Fe and Al.	Coagulant: Fe(III): 72% and Al(III): 27.5%, low concentrations of Mn, Zn, Ca, Mg.	[33]
Waste Li foils and expired ferrous sulfate granules	2 wastes type: Li from spent CR2025 Li-coin-cells and expired ferrous sulfate granules collected from the households, hydrothermal preparation for LiFePO_4_/C nanoparticles cathode.	LiFePO_4_/C cathode.	[35]
FeSO_4_·7H_2_O from TiO_2_ industrial production.	Premixing of two solids (NaOH with FeSO_4_·H_2_O) for single solid production as iron sulfate coated with alkali hydroxides (80 to 90 g of Fe/kg of iron), oxidation with diluted chlorine in the fluidized bed. Fe(VI): 30–55%.	Alkali ferrates ~1 mm (A_2_FeO_4_, A: Na, K) with Fe(VI), for cleaning waters, wastewater, and other effluents.	[30]
MS and blast furnace flue dust	MS and BF dust dried at 105 °C for 12 h, mixed with Portland cement as binder and water, 3 different C/O mass ratios (0.25, 0.5, and 0.75), high-temperature and reducing atmosphere conditions.	Cylindrical briquettes for blast furnaces. Average weight and height: 2.7 ± 0.02 g and 10 ± 0.1 mm.	[6]
MS for colored ceramic pastes	1, 3, 5, and 10 wt.% MS pretreatments: milling, 212 µm sieving, maximum firing temperature (1043 ± 1165 °C).	Dark grey hue ceramic pastes with MS incorporation.	[1]
Spent pickle liquor from steel manufacturers	MS leaching with H_2_SO_4_ (at concentrations of 5%, 10%, and 15%), liquor sample rich in Fe^2+^ formation, 80 °C evaporation, and crystallization of ferrous sulfate.	Melanterite (FeSO_4_⋅7H_2_O) main compound, szomolnokite (FeSO_4_⋅H_2_O), Rozenite (FeSO_4_⋅4H_2_O).	[63]
Spent pickling liquors from a steel surface treatment factory	Ultrasonic-assisted chemical co-precipitation, molar ratios of Fe(III)/Fe(II), pH 0.3–0.5, Fe total 105.6 g/L, HCl 10.6 g/L, heavy metals traces, suspended solids (SS) filtration, alkaline buffer solution (pH 13) add, reaction time: 20–30 min, separation by sedimentation, washing solid, ultrasonication.	Fe_3_O_4_ cubic nanoparticles, 13–23 nm diameter, super-paramagnetic.	[64]
Ferrous sulfate waste from TiO_2_ production	Ferrous sulfate reductive decomposition with pyrite, reaction temperature: 580–770 K. The desulfurization rate: 98.55%.	Fe_3_O_4_ nano-sized and SO_2_.	[65]
MS iron waste	Fe(II) and Fe(III) precursors from raw MS leached with H_2_SO_4_ conc, heating to dryness, solid products used as starting material. Fe_3_O_4_: 10 g Fe(II) in 120 mL distilled water, 25% NH_4_OH, pH 11–12, room temperature, 20 h, black precipitate/γ Fe_2_O_3_: 200 °C thermal treatment of Fe_3_O_4_/α FeO(OH): 20 g Fe(III) in 500 mL distilled water, 1 M NaHCO_3_, pH: 5–7, 100 °C, 1 h, room temperature/α Fe_2_O_3_: 600–900 °C calcination of α FeO(OH), 5 h.	Fe_3_O_4_, α and γ Fe_2_O_3_, α FeO(OH) (<0.1 mm), high surface area, various colors orange–brown, brown–red, bright red, maroon, purple and gray.	[66]
MS samples	Raw samples pretreatment: attrition milling, sieving, aqua regia digestion: sodium borohydride (NaBH_4_) and NaOH solution as pH stabilizer: 30–40 nm NPs formation. cetyltrimethylammonium bromide (CTAB) as a cationic surfactant, butanol as cosurfactant, octane as non-aqueous oil phase: 5 nm Fe spherical NPs formation. Hydrazine as stable reducing reagent: 5 nm body-centered cubic Fe NPs.	Nanoscale zerovalent Fe (nZVI), between 5 and 40 nm.	[67]

**Table 2:** Efficiencies regarding pollutant removal applying valorized ferrous wastes.

Pollutant	Waste Type	Application, Efficiency	References
Solid residues from air pollution control system (APC)	FeSO_4_ from TiO_2_ production as coagulant.	Leaching tests: liquid–solid ratio (L/S): 3 L/kg, stirring, aeration 24 h. 84–123% removal efficiencies for salts (Cl, K, Na); ≤0.001% for heavy metals (Pb: 27–39 μg/L, Cd: 2.6–4.6 μg/L), and 1–5% Cr.	[165]
N-NH_3_, COD	FeSO_4_⋅7H_2_O waste from the TiO_2_ manufacturing industry as a coagulant.	SRPE: 98.19% and 93.86% removal efficiencies for NH_3_-N and COD, 70 min, 900 mg/L coagulant doses, 62 °C.	[32]
pH, suspended solids, turbidity, color, conductivity, metals, hardness, sulfate	poly-alumino-iron sulfate (PAFS) as a coagulant based on iron and aluminum recovered from AMD.	1000 mL water sample, 0.4 mM (Fe + Al) PAFS-SP/AMD, pH 7.0, 100 rpm, 5 min, 10 min solids settling. All analyzed pollutants were removed and concentrations were under Brazilian standards for drinking water.	[33]
As(V)	Fe NPs from local waste materials: iron-coated sand, cast iron filings, steel wool, amended blast furnace slags.	As(V) adsorption from sludge or contaminated water.	[34]
As(V) and As(III)	Cast-iron filings (wastes from mechanical workshops, lathes) and steel wool (commercially available, used for cleaning wood surfaces prior to polishing).	90–95% As removal, increased with increased sorbent dosage from 2 to 20 g/L pH favorable conditions, data fitted with the Langmuir model, sorption reduced progressively from pH 3.0 to 9.0 and decreased beyond 9.0.	[21]
As(V)	Fe(III)/Cr(III) hydroxide sludge as waste: Cr(VI) as a corrosion inhibitor in cooling water and electrolytic Fe(II) as a reductive agent for Cr(VI) to Cr(III), pH acid.	As(V) adsorption followed a first-order rate independent of pH (3–10). Desorption with NaOH solutions. pH 4, 20–100 mg/L As, 500 mg waste/50 mL aqueous solution, 5 h, 32 °C, 11.02 mg/g.	[22,23,166]
4-chlorophenol (4-CP)	Graphite-supported zero-valent iron–copper bimetallic catalyst (ZVI-Cu/C): MS waste with spent lithium-ion battery (LIB) anode by carbothermic reduction, 1:4 mass ratios.	4-CP degradation on the catalyst in water (reduction and heterogeneous Fenton reactions). Spent LIB anode powders (0.5 g, size < 0.15 mm) mixed with MS (0.5, 1.0, 1.5, 2.0 g) of MS, 1000 °C, 2 h, N_2_ atmosphere (120 cm^3^·min^−1^) in a tubular furnace; 100% degradation with higher Fe in ZVI-Cu/C.	[167]
Cr(VI)	Scrap with iron air-formed oxides on surfaces.	Batch system, aqueous solutions, pH: 2.10–7.10, temperature: 10–40 °C, Cr(VI): 19.2–576.9 M.	[149]
Cd, Ni, Cu	MS as a precursor for Fe_3_O_4_, γ Fe_2_O_3_ NPs.	≥90% removal efficiency, after 10 min, Langmuir isotherm data.	[168]

## Data Availability

Not applicable.
